# Effects of Vertical Sleeve Gastrectomy on Weight Loss, Eating Behaviors, and Weight Concern Eight Months Postsurgery

**DOI:** 10.7759/cureus.62383

**Published:** 2024-06-14

**Authors:** Maxime Legendre, Andrée-Anne Guénette, Alycia Jobin, Catherine Bégin

**Affiliations:** 1 School of Psychology, Laval University, Quebec City, CAN

**Keywords:** dietary restraint, obesity, gastrectomy, food addiction, eating disorder, bariatric surgery

## Abstract

Objectives: Following vertical sleeve gastrectomy (VSG), the role of eating behaviors in weight regain remains unclear. This study aimed to examine the effects of VSG on excess weight loss (EWL) and five eating-related variables (food addiction, disinhibition, susceptibility to hunger, dietary restraint, and weight concern) while exploring their associations before and eight months post-surgery.

Materials and methods: A sample of 76 participants who underwent VSG was recruited from a healthcare center in Quebec, Canada. Measurements included body mass index (BMI), the Eating Disorder Examination (weight concern), the Yale Food Addiction Scale (food addiction), and the Three-Factor Eating Questionnaire (disinhibition, susceptibility to hunger, and dietary restraint). T-tests were conducted between pre-surgery (T0) and eight-month post-surgery (T8), and correlations were examined between T0 and T8, within T0, and within T8.

Results: The mean EWL was 63.43% ± 13.14 at T8. Comparisons between T0 and T8 showed a significant decrease in food addiction, disinhibition, and susceptibility to hunger (p = 0.001-0.005). No significant differences were observed for dietary restraint and weight concerns. BMI at T0 was negatively correlated with EWL at T8 (r = -0.45). Within T0, a negative correlation was observed between food addiction and dietary restraint (r = -0.42), which changed from negative to positive within T8 (r = 0.35).

Conclusions: This study confirmed that VSG is effective for weight loss and associated with a reduction in maladaptive eating behaviors. Postsurgery, individuals with greater food addiction exhibited more dietary restraint, suggesting a need for restraint among those experiencing a strong drive toward food. However, weight concerns remained high even after significant weight loss, indicating that weight loss alone may not be sufficient for change. A postsurgery medical follow-up focusing on overall well-being and lifestyle adaptation would be a crucial complement.

## Introduction

Obesity, characterized by a body mass index (BMI) ≥ 30 kg/m^2^, stands as one of the foremost health challenges worldwide [1]. In Canada, approximately 27% of the population falls within the obesity class [2], predisposing them to heightened risks of cardiovascular disease, sleep apnea, type II diabetes, musculoskeletal disorders, certain cancers, and psychiatric disorders [1,3,4]. Notably, risk also increases with higher BMI, and severe obesity (BMI ≥ 40) is associated with a substantially elevated risk of developing such complications [1,5]. Furthermore, severe obesity is the weight category with the swiftest progression in recent years [6]. Standard interventions for both obesity and severe obesity primarily target weight loss through dietary adjustments and increased physical activity, yet their efficacy remains limited. For instance, a recent meta-analysis including 1,180 randomized controlled trials concluded that, while a majority of studies demonstrated significant weight loss within six to 12 months, the magnitude of loss generally amounted to less than 2 kg [7].

Currently, the most effective intervention for weight loss among individuals with obesity is bariatric surgery [8]. In Canada, the practice of bariatric surgery is on the rise, with nearly 10,500 procedures conducted in 2017-2018 [9]. In the United States, the National Institutes of Health (NIH) suggests considering bariatric surgery for individuals with a BMI ≥ 40, or a BMI ≥ 35 coupled with comorbidities such as type II diabetes, sleep apnea, or cardiovascular disease [10]. Bariatric surgery is defined as a transformation/modification of the stomach with or without modification of the intestine to achieve weight loss [10]. The efficacy criteria for bariatric surgery were established by Reinhold [11] and are based on excess weight loss (EWL) percentage calculated according to the following formula: ((presurgery BMI - postsurgery BMI) / (presurgery BMI - 25)) x 100. EWL is classified as excellent if surpassing 75%, satisfactory if falling between 50% and 75%, moderate if between 25% and 50%, and deemed a failure if below 25% [11]. Although various types of bariatric surgery exist, vertical sleeve gastrectomy (VSG) is the most popular procedure in Canada and worldwide [12,13]. VSG entails the removal of approximately two-thirds of the stomach, leaving behind a smaller, sleeve-like gastric pouch capable of holding less than 200 mL, and primarily operates through gastric volume restriction and modulation of appetite-regulating hormones [14]. Consequently, individuals undergoing VSG experience reduced food intake and heightened satiety. Additionally, the removal of the gastric fundus, which houses ghrelin-secreting cells, contributes to diminished hunger sensations [14].

More and more studies are documenting the effectiveness of VSG in the short term. Generally, results suggest that EWL is around 50% (range: 40-65%) at 12 months postsurgery [15,16]. In addition to significant weight loss, studies suggest improvement in several eating-related variables. Among others, studies report a reduction in weight, shape, and eating concerns after VSG [17]. Additionally, a reduction in disinhibition (i.e., overconsumption of food in response to stimuli other than hunger) and susceptibility to hunger (i.e., hypersensitivity/reactivity to internal sensations and external cues associated with hunger, leading to food intake) have been reported at 12 and 48 months postsurgery [18,19]. Regarding dietary restraint, some studies report no significant change at 12 and 48 months postsurgery [18,20], while others report an increase at 19 months postsurgery [19]. Finally, a reduction in food addiction (FA) has also been documented by various studies [21-23]. FA is characterized by the same symptoms as alcohol and other substance use disorders but with food as the object of addiction. Although not a recognized disorder in the Diagnostic and Statistical Manual of Mental Disorders (DSM-5) [24], FA has generated significant interest since some researchers believe it may explain weight regain after surgery. For instance, in a study including 44 patients, eight of whom received VSG, FA remission was observed at nine months postsurgery in 93% of participants identified with presurgery FA. The investigators reported that an initial weight loss of 20% resulted in a significant reduction in FA [22]. In another study, including 166 patients of whom 136 received VSG, the prevalence of FA fell from 57.8% presurgery to 7.2% and 13.7% at six and 12 months postsurgery, respectively [23].

Because of possible weight regain, it is important to assess early changes in eating behaviors and how those behaviors could predict long-term weight management success [25,26]. According to a review of the literature, rates of weight regain in VSG patients range from 5.7% at two years to 75.6% at six years [26]. This review of predictors of weight regain after VSG raises the possibility that weight regains may be attributable to surgery-related factors, such as sleeve dilation and increased ghrelin levels, or to individual-related factors, such as the resumption of maladaptive eating behaviors [26]. Specifically, a higher pre-surgery BMI appears to be to most important factor associated with poorer efficacy of VSG [27,28]. Regarding postsurgery factors, a multitude of eating-related variables have been more or less consistently associated with poorer efficacy of VSG [29,30]. Finally, although several predictors have been studied separately, no study has attempted to understand how behavioral patterns (i.e., behaviors associated with each other) might be influenced by VSG.

The overall aim of the study was to better understand the relationship between VSG and different eating-related variables. The first objective was to examine the short-term effects of VSG (eight months postsurgery) on weight loss and five eating-related variables: FA, disinhibition, susceptibility to hunger, dietary restraint, and weight concern. An EWL of 50% or more was expected, as well as a significant decrease in all eating-related variables, except dietary restraint. The second objective was to examine associations between EWL and eating-related variables presurgery and eight months postsurgery. Presurgery, it was expected that only BMI would be negatively associated with EWL. Postsurgery, no hypothesis was postulated, given the inconsistency found in the literature. Finally, associations between pre- and postsurgery eating-related variables were analyzed for exploratory purposes.

## Materials and methods

Participants

A total of 175 patients eligible for bariatric surgery were recruited at the Quebec Heart and Lung Institute (IUCPQ) from January 2014 to June 2016. To be included, patients had to (1) have severe obesity (BMI ≥ 40 kg/m^2^ or ≥ 35 kg/m^2^ with at least one comorbidity), (2) receive VSG, and (3) be 18 years of age or older. Patients reporting (1) substance addiction or (2) pregnancy were excluded. The sample included 55 women and 21 men (n = 76), predominantly White (98.7%), with a mean age of 41.01 years ±7.10 (23-58) and a mean presurgery BMI of 48.13 kg/m^2^ ±7.74 (range: 36.60-75.50). Weight-loss data were collected for 76 participants (100%) presurgery and 57 participants (75%) eight months postsurgery. Questionnaires were completed by 52 participants (68%) presurgery and 29 participants (38%) eight months postsurgery.

Procedure

During their presurgery medical appointment at the IUCPQ, a research nurse met with patients to present the research project and verify their eligibility. Following this appointment, patient volunteers were contacted again and met by a research assistant to obtain informed signed consent and conduct a semi-structured interview to collect eating behaviors and attitudes. Finally, patients were given a battery of questionnaires to complete at home.

Prior to their postsurgery follow-up appointment at the IUCPQ (eight months postsurgery), participants were recontacted by telephone. During this call, the research assistant verified the patients' interest in the second phase of the study and administered the same semi-structured interview. Next, the same battery of questionnaires, including written informed consent, was mailed to patient volunteers. Finally, at the follow-up appointment at the IUCPQ, the research assistant collected the completed questionnaires.

Participants' height and weight were measured by the IUCPQ medical staff at the eight-month postsurgery follow-up. This information was transmitted to the investigators with the participants' consent. The IUCPQ Research Ethics Board (BCÉR) approved the study.

Measures

A demographic questionnaire was used to collect information such as age, gender, ethnicity, employment status, family situation, and education level. Participants' height, weight, and BMI were collected by the IUCPQ medical staff.

Food Addiction (FA)

The Yale Food Addiction Scale (YFAS) [31,32] is a self-report questionnaire designed to assess behaviors, thoughts, and emotions related to FA over the past 12 months, using 25 items based on the seven DSM-IV-TR diagnostic criteria for substance use disorder [33]. Items are answered on a dichotomous scale (yes or no) and on a 5-point Likert scale ranging from zero (never) to four (more than four times a week or every day). The endorsement of at least three of the seven criteria plus the significant distress or functional impairment criterion, gives an unofficial FA diagnosis, and the number of criteria endorsed (from zero to seven) gives a measure of severity. The seven criteria are (1) eating more or longer than desired, (2) inability to stop or reduce, (3) much time spent eating, (4) abandonment of social activities, (5) withdrawal, (6) physical or emotional consequences, and (7) tolerance. This questionnaire showed good test-retest reliability (kappa = 0.73) for the diagnosis of FA [34] and good internal consistency in its original version (α = 0.86) [31] and in the present study (α = 0.87).

Disinhibition, Susceptibility to Hunger, and Dietary Restraint

The Three-Factor Eating Questionnaire (TFEQ) [35] is a self-report questionnaire designed to measure three types of eating behaviors: disinhibition, susceptibility to hunger, and dietary restraint. Disinhibition targets the overconsumption of food in response to stimuli other than hunger (16 items; e.g., Sometimes when I start eating, I just can’t seem to stop), while susceptibility to hunger targets the tendency to consume food in the presence of hunger sensations and perceptions (14 items; e.g., I often feel so hungry that I just have to eat something), and dietary restraint targets the control of food intake (21 items; e.g., I deliberately take small helpings as a means of controlling my weight). Items are answered on a dichotomous scale (true or false) and on a 5-point Likert scale ranging from one (low endorsement) to four (strong endorsement). The sum of the items gives a severity score. This questionnaire showed adequate test-retest reliability ranging from r = 0.53 to 0.86 [36] and good internal consistency for each of the subscales, both in the original validation study (KR-20 from 0.85 to 0.93) [37] and in the present study (α = 0.71-0.84).

Weight Concern

The Eating Disorder Examination (EDE) [38] is a semi-structured interview designed to assess the presence of an eating disorder (anorexia nervosa, bulimia nervosa, or binge eating disorder). The EDE targets behaviors, thoughts, and concerns present over the past three months and comprises four sections: restraint, weight concern, shape concern, and eating concern. The higher the scores, the greater the frequency or severity. Only the weight concern section was used in the present study because the four sections showed high collinearity. The EDE is a widely used interview validated with several populations with good test-retest reliability (r = 0.51-0.74 for weight concern) [39,40]. In the present study, the weight concern subscale showed good internal consistency (α = 0.78).

Statistical analysis

Statistical Product and Service Solutions software (SPSS, 24.0; IBM SPSS Statistics for Windows, Armonk, NY) was used for descriptive analyses, mean comparisons, and correlations. BMI and the five eating-related variables underwent testing for normal distribution and the identification of univariate and multivariate outliers. No data transformation was necessary, and no participants were removed. BMI and the five eating-related variables were measured presurgery (T0) and eight months postsurgery (T8). EWL was calculated using the following formula: ((BMI T0 - BMI T8) / (BMI T0 - 25)) x 100. Then, paired sample t-tests were performed to assess significant differences between T0 and T8 for the five eating-related variables. Pearson correlations were performed between EWL and the five eating-related variables at T0 and T8. Post-hoc partial correlation was performed between EWL at T8 and presurgery BMI with age, sex, and annual household income as covariables.

## Results

Demographic characteristics and presurgery BMI (T0) of participants who completed the questionnaires at T0 are presented in Table 1. Table 2 shows the comparisons of participants who completed the questionnaires at T0 and T8 with those who completed them at T0 only. Except for age, participants who dropped out did not differ from those who completed T8 in either demographic characteristics or eating-related variables.

**Table 1:** Presurgery demographic characteristics and BMI (n = 52). BMI = Body Mass Index

	Mean (SD)	%
Age, mean (SD)	40.90 (7.14)	
BMI, mean (SD)	46.95 (6.55)	
Gender (%)		
Women		76.9%
Men		23.1%
Race (%)		
White		98.7%
Latino		1.3%
Employment status (%)		
Full-time worker		76.5%
Part-time worker		7.8%
Unemployed		15.7%
Marital status (%)		
Married/common-law		80.8%
Single		15.4%
Divorced/separated		3.8%
Education level (%)		
University degree		25.5%
Collegial or professional training		35.1%
High school diploma or less		39.4%
Annual household income (%)		
Less than $40 000 CAD		24.0%
$40 000 to $79 999 CAD		31.0%
$80 000 CAD and over		45.0%

**Table 2:** Comparisons between those who completed T8 and those who dropped out. BMI = Body Mass Index; T8 = Eight Months Postsurgery ^a ^Number of food addiction symptoms

	Completed (n = 29)	Dropped out (n = 23)	t(df)	χ²	p
Age, mean (SD)	42.90 (5.98)	38.39 (7.80)	t(50) = -2.36		0.022
BMI, mean (SD)	46.70 (6.32)	47.26 (6.96)	t(50) = 0.30		0.764
Gender (%)				χ² = 1.26	0.262
Women	82.8%	69.6%			
Men	17.2%	30.4%			
Race (%)				χ² = 1.11	0.293
White	100.0%	95.7%			
Latino	0.0%	4.3%			
Employment status (%)				χ² = 8.29	0.218
Full-time worker	69.0%	87.0%			
Part-time worker	13.8%	0.0%			
Unemployed	17.2%	13.0%			
Marital status (%)				χ² = 1.93	0.381
Married/common-law	75.9%	69.0%			
Single	6.9%	10.3%			
Divorced/separated	17.2%	0.0%			
Education level (%)				χ² = 4.02	0.404
University degree	24.1%	20.7%			
Collegial or professional training	31.0%	34.5%			
High school diploma or less	44.8%	24.1%			
Annual household income (%)				χ² = 3.75	0.711
Less than $40 000 CAD	19.2%	26.1%			
$40 000 to $79 999 CAD	30.8%	30.4%			
$80 000 CAD and over	50.0%	43.5%			
Eating-related variables, mean (SD)					
Food addiction (/11)^a^	2.41 (1.50)	3.22 (2.04)	t(50) = 1.64		0.108
Disinhibition (/16)	7.97 (2.99)	8.30 (3.69)	t(50) = 0.37		0.716
Susceptibility to hunger (/14)	5.03 (3.64)	6.09 (3.70)	t(50) = 1.03		0.309
Dietary restraint (/21)	9.04 (4.80)	8.57 (4.76)	t(50) = -0.53		0.597
Weight concern (/6)	3.93 (2.27)	3.22 (1.93)	t(50) = -1.20		0.235

Effects of VSG on EWL and eating-related variables

The mean EWL was 63.43% ± 13.14 (41.11-89.78) at T8 (n = 57). Comparisons between T0 and T8 showed a significant decrease in FA, disinhibition, and susceptibility to hunger (Table 3). For dietary restraint and weight concern, no significant difference was observed.

**Table 3:** Comparisons between T0 and T8 for eating-related variables (n = 29).

	T0	T8	t(df)	p
Food addiction (/11)	2.41 (1.50)	1.34 (0.86)	t(28) = 3.50	0.002
Disinhibition (/16)	7.97 (2.99)	5.38 (2.76)	t(28) = 3.92	0.001
Susceptibility to hunger (/14)	5.03 (3.64)	2.79 (2.98)	t(28) = 3.04	0.005
Dietary restraint (/21)	9.04 (4.80)	10.44 (3.90)	t(28) =-1.64	0.113
Weight concern (/6)	3.93 (2.27)	3.72 (1.62)	t(28) = 0.45	0.657

Associations between EWL and eating-related variables

Only two significant correlations were observed between T0 and T8, a moderate negative correlation between BMI at T0 and EWL at T8 (r = -0.45, p < 0.001) and a moderate positive correlation between dietary restraint at T0 and T8 (r = 0.49, p = 0.009; Table 4). The negative correlation between BMI at T0 and EWL at T8 remains significant after controlling for age, sex, and annual household income (r = -0.43, p = 0.010). Within T0, moderate-to-strong positive correlations were observed between FA and disinhibition (r = 0.63, p < 0.001), FA and susceptibility to hunger (r = 0.55, p < 0.001), and disinhibition and susceptibility to hunger (r = 0.73, p < 0.001). Moderate negative correlations were observed between FA and dietary restraint (r = -0.42, p = 0.002) and between susceptibility to hunger and dietary restraint (r = -0.31, p = 0.026). Within T8, a strong positive correlation between disinhibition and susceptibility to hunger (r = 0.71, p < 0.001) was found, and the moderate correlation between FA and dietary restraint changed from negative to positive (r = 0.35, p = 0.048).

**Table 4:** Correlations between T0 and T8 for EWL and eating-related variables. T0 = Pre-surgery; T8 = Eight Months Postsurgery; EWL = Excess Weight Loss * p < 0.05, ** p < 0.01

	1	2	3	4	5	6	7	8	9	10	11	12
1. EWL at T8	1	-0.45**	-0.15	-0.02	0.05	-0.02	-0.24	0.29	-0.20	-0.08	0.24	0.08
Presurgery (T0)												
2. BMI		1	0.14	0.11	-0.01	-0.08	0.17	-0.13	-0.06	0.02	-0.15	-0.21
3. Food addiction			1	0.63**	0.55**	-0.42*	0.18	0.11	0.08	0.08	-0.11	0.28
4. Disinhibition				1	0.73**	-0.26	0.12	0.35	0.24	0.12	0.03	0.17
5. Susceptibility to hunger					1	-0.31*	0.09	0.16	0.04	0.29	0.01	0.25
6. Dietary restraint						1	-0.02	0.02	-0.08	-0.08	0.49*	-0.16
7. Weight concern							1	0.20	0.04	0.16	0.26	0.22
Eight-month post-surgery (T8)												
8. Food addiction								1	0.12	0.04	0.35*	0.20
9. Disinhibition									1	0.71**	0.04	-0.21
10. Susceptibility to hunger										1	0.22	-0.13
11. Dietary restraint											1	-0.11
12. Weight concern												1

## Discussion

The main objective of this study was to explore the short-term effects of VSG on weight loss and different eating-related variables. Results revealed a mean EWL of 63% at eight months postsurgery, surpassing the widely used criterion of EWL > 50% for bariatric surgery success assessment [11]. This result aligns with EWL percentages ranging from 60% to 68% reported in similar studies at six months postsurgery [41-43], as well as percentages ranging from 55% to 80% at 12 months postsurgery [12,44]. Regarding eating-related variables following VSG, overall improvements were observed. Participants exhibited reduced FA, disinhibition, and susceptibility to hunger. However, dietary restraint and weight concerns remained unchanged.

The decrease in FA symptoms at eight months postsurgery is consistent with the results from previous studies on the effects of various surgical procedures, including VSG, at time points ranging from six to 12 months postsurgery [22,23]. Three studies investigated the effect of VSG on eating behaviors measured by the TFEQ (disinhibition, susceptibility to hunger, and dietary restraint) at measurement times ranging from 12 to 48 months postsurgery [18-20]. Consistent with our findings, these studies converge on a decrease in disinhibition and susceptibility to hunger without significant changes in dietary restraint. Despite significant weight reduction at eight months postsurgery, participants continued to express concerns about their weight. Indeed, both pre- and postsurgery levels of weight concern (3.93 and 3.72, respectively) exceeded values observed in individuals with binge eating disorder (M = 2.7±2.3) and were comparable to those observed in individuals with bulimia (M = 4.3±2.0) and anorexia (M = 3.7±2.3) [45]. These results stand in contrast to previous studies that reported a decrease in weight concerns up to 48 months after VSG [18,46]. This discrepancy could be attributed to the difference in time interval. In the first eight months postsurgery, most patients experience a period of progressive changes characterized by rapid weight loss, followed by a deceleration phase with potential fluctuations. Therefore, concerns may arise due to a slowdown in weight loss or, alternatively, the lack of stability in weight and body image. Over the longer term, patients may experience increased stability in weight and body image, potentially alleviating their concerns.

The second objective aimed to investigate the relationship between EWL and eating-related variables. None of the presurgery eating-related variables were associated with EWL eight months postsurgery, consistent with an emerging trend in the literature indicating that presurgery eating-related variables are not related to weight loss outcomes, regardless of the surgical procedure [19,20,23,28]. Similarly, none of the eating-related variables at eight months postsurgery were associated with EWL. This suggests that eating behaviors during the first eight months post-surgery have minimal associations with weight loss outcomes, aligning with findings from prior studies conducted at various follow-up intervals after VSG [18-20,23,46]. Presurgery BMI emerged as the sole factor negatively associated with EWL, indicating that the higher the BMI presurgery, the lower the EWL eight months postsurgery, even when controlling for age, sex, and annual household income. A review of the literature examining predictors of success (EWL > 50%) following bariatric surgery revealed that presurgery BMI was negatively associated with weight loss by 37 of the 62 studies analyzed [27,28]. Overall, these results suggest that pre-surgery BMI serves as the primary predictor of short-term success following VSG.

Regarding the relationships between eating-related variables, distinct patterns emerged pre- and postsurgery. Firstly, FA, disinhibition, and susceptibility to hunger were positively correlated in pre-surgery, indicative of a generalized pattern of overeating. While the association between disinhibition and susceptibility to hunger persisted postsurgery, correlations with FA were absent. Furthermore, FA (and to a lesser extent, susceptibility to hunger) displayed a negative association with dietary restraint presurgery. However, eight months postsurgery, the relationship between FA and dietary restraint reversed while maintaining a similar magnitude. Therefore, presurgery, higher FA was associated with less dietary restraint, whereas, postsurgery, it was associated with increased dietary restraint. To interpret this finding, it is important to consider that FA decreased significantly following VSG, contrasting with the relatively stable dietary restraint observed. Without surgical intervention, FA (and possibly other overeating behaviors) could be experienced and reported as a consequence of an inability to restrain. Consequently, a poor ability to restrain (low dietary restraint) would be associated with high FA. Conversely, as FA decreases postsurgery, the reported dietary restraint may represent the perceived need for restraint. Those for whom FA has decreased no longer feel the need to restrain themselves, whereas those with lingering FA may perceive a higher need for restraint. A neurological hypothesis could elucidate this positive relationship. Researchers investigating responses to food cues (e.g., odor) found a positive correlation between dopamine responsiveness and cognitive restraint, suggesting that heightened reactivity to food cues may drive increased restraint efforts [47].

There are a number of limitations that should be noted in this study. Firstly, the small sample size, coupled with attrition, makes it difficult to generalize the results obtained to the general bariatric population. Attrition rates in adult bariatric cohorts are commonly high, ranging from 25% to 63% [48,49], depending on the surgery procedure and the nature and length of post-surgery follow-up. Consequently, replication of these findings with a larger sample is essential. Secondly, the sample was under-represented with men and almost exclusively White. Replication with a more diverse sample is needed. Thirdly, the study design precludes determining whether the observed reduction in eating behaviors post-surgery reflects genuine lifestyle changes or mere consequences of surgical anatomical alterations. This limits the scope of the present study's conclusions and recommendations. A randomized assignment with a post-surgery control group would address this limitation.

## Conclusions

This study underscores the efficacy of VSG as an intervention for weight loss, accompanied by reductions in certain maladaptive eating behaviors. In the longer term, it will be important to verify whether the effects observed persist as the physiological mechanisms of VSG weaken. Notably, three findings hold significant implications for clinicians working with these patients. Firstly, presurgery BMI emerged as the sole factor negatively associated with EWL, indicating that the higher the BMI presurgery, the lower the EWL eight months postsurgery. Secondly, the shift from a negative to a positive correlation between FA and dietary restraint postsurgery suggests that individuals with greater FA exhibit increased dietary restraint, possibly indicating a greater need for restraint rather than objectively high restraint levels. Thirdly, weight concern was still high even after significant weight loss, suggesting that weight loss alone is not sufficient to generate change. Postsurgery, patients will need to adapt to their new body and change their perception of themselves, which for many has been embedded since childhood. Furthermore, this finding may reflect a fear of losing control of their body and regaining the weight they lost. Postsurgery medical follow-up focusing on holistic well-being and lifestyle adjustments, rather than solely weight maintenance, is crucial to support these patients effectively.
